# The bacterial Mre11–Rad50 homolog SbcCD cleaves opposing strands of DNA by two chemically distinct nuclease reactions

**DOI:** 10.1093/nar/gky878

**Published:** 2018-10-02

**Authors:** Jan-Hinnerk Saathoff, Lisa Käshammer, Katja Lammens, Robert Thomas Byrne, Karl-Peter Hopfner

**Affiliations:** 1 Department of Biochemistry, Ludwig-Maximilians-Universität München, Feodor Lynen Straße 25, 81377 Munich, Germany; 2 Gene Center, Ludwig-Maximilians-Universität München, Feodor Lynen Straße 25, 81377 Munich, Germany; 3 Center for Integrated Protein Science, Munich, Germany

## Abstract

The Mre11–Rad50 complex is a DNA double-strand break sensor that cleaves blocked DNA ends and hairpins by an ATP-dependent endo/exonuclease activity for subsequent repair. For that, Mre11–Rad50 complexes, including the *Escherichia coli* homolog SbcCD, can endonucleolytically cleave one or both strands near a protein block and process free DNA ends via a 3′-5′ exonuclease, but a unified basis for these distinct activities is lacking. Here we analyzed DNA binding, ATPase and nuclease reactions on different DNA substrates. SbcCD clips terminal bases of both strands of the DNA end in the presence of ATPγS. It introduces a DNA double-strand break around 20–25 bp from a blocked end after multiple rounds of ATP hydrolysis in a reaction that correlates with local DNA meltability. Interestingly, we find that nuclease reactions on opposing strands are chemically distinct, leaving a 5′ phosphate on one strand, but a 3′ phosphate on the other strand. Collectively, our results identify an unexpected chemical variability of the nuclease, indicating that the complex is oriented at a free DNA end and facing a block with opposite polarity. This suggests a unified model for ATP-dependent endo- and exonuclease reactions at internal DNA near a block and at free DNA ends.

## INTRODUCTION

The maintenance and accurate replication of genomes are fundamental processes in all kingdoms of life. Genome integrity is challenged by DNA damage caused by a large variety of physical, chemical and biochemical activities. DNA damage and complications in DNA replication can cause genomic alterations ranging from point mutations to gross chromosomal aberrations and aneupleuidy, which in humans is associated with the development of cancer and other diseases. In all phylogenetic kingdoms the propagation and maintenance of the genome critically depends on various pathways that detect, signal and repair DNA damage and deal with replicative stress (1).

The nuclease Mre11 and the ATPase Rad50 form an evolutionary highly conserved complex, which is involved in genome maintenance and replication by detecting and processing DNA double-strand breaks, hairpins and other abnormal terminal DNA structures (2). The bacterial homologs are known as SbcC (ATPase) and SbcD (nuclease) and form the SbcCD complex (3). The eukaryotic complexes contain a third subunit, Nbs1 in mammals and Xrs2 in yeast, and are denoted MRN or MRX (2). MRN/X and SbcCD detect DNA end structures and can process blocked or obstructed DNA ends and hairpins to make them accessible for DSB repair (4–8). The main DSB repair pathways following DNA end processing by MRN/X are various end joining reactions and homologous recombination (HR) (9,10).

MRN has a variety of biochemical activities. It displays 3′-5′ dsDNA exonuclease and ssDNA endonuclease activities, and opens hairpins in the presence of ATP (4,11–13). However, the physiologically most critical activity appears to be an ATP hydrolysis-dependent 5′ endonuclease activity at a 15–25 bp distance from blocked DNA ends, followed by limited 3′-5′ resection towards the DNA end (14–18). The endonucleolytic incision is essential to remove covalent DNA–protein crosslinks (DPCs), such as those formed by abortive topoisomerases in cycling cells or by the topoisomerase-like Spo11 during meiosis (8,19). MRN/X is also capable of removing the DSB binding factor Ku from DNA ends prior to HR (20–23). The mechanism of sensing of blocked ends by MRN/X or SbcCD is unclear, but recent studies show that MRN can bind internal sites of DNA and slide towards blocked DNA ends (24).

Like MRN/X, SbcCD has 3′-5′ exonuclease activity, cuts the DNA near protein-bound DNA ends and cleaves hairpin structures 5′ of the loop (7,25). *In vivo* studies revealed that SbcCD cleaves covalently bound topoisomerases from DNA and removes specific DNA-secondary structures, including hairpins and cruciform structures (3,26,27). This role is conserved in budding yeast, where the Mre11 nuclease activity is essential to open hairpin structures and prevent the formation of palindromic duplications (6,28). More recent studies showed that SbcCD is critical in enabling proper replication termination by processing DNA bridges between the duplicated chromosomes that arise after the two convergent replication forks have passed each other (29).

Whereas the uncapping of hairpins and de-blocking of protein-bound DNA ends appears to be an evolutionarily conserved biochemical activity, pro- and eukaryotic complexes also show some differences. MRN/X is much more regulated through Nbs1/Xrs2 and requires the additional factor CtIP/Sae2/Ctp1 for end resection (30,31). In contrast, SbcCD possesses intrinsic, robust endonuclease activity by itself and can shorten the DNA ends further through an ATP-dependent binary endonuclease activity that cleaves both DNA strands, introducing serial DNA double-strand breaks in ∼10 bp intervals (32). This endonucleolytic cleavage of both strands has been recently reported for MRN, suggesting that even the complete clipping of blocked DNA termini is an evolutionarily conserved, inherent activity of the complexes (33).

Mre11/SbcD forms a dimer *via* two protein phosphatase 2 family phosphodiesterase/nuclease domains (34), and additionally contains a DNA-binding ‘capping’ domain (35,36), a linker, and a Rad50 binding domain (RBD) (37). SbcC/Rad50 contains an ATP-binding cassette (ABC) type nucleotide binding domain (NBD) with a 15–50 nm long antiparallel coiled-coil insertion that is capped by a zinc-hook dimerization motif (38). Two Mre11 and two Rad50 monomers assemble with a globular DNA binding and processing head module, containing the Mre11 dimer and two Rad50 NBDs, and a rod or ring-like protrusion that is formed by the two coiled-coils (34,39,40). Structural studies revealed that the ATP-dependent dimerization of the Rad50 NBD is coupled to the binding of ∼20 bp of DNA (41,42). However, previous studies have failed to provide a mechanism for how MRN/SbcCD detects DNA ends, let alone how it processes them in an ATP-dependent manner. In the crystal structure of the ATP bound and ATP/DNA bound conformations of Rad50, Mre11’s DNA binding cleft and nuclease active site are blocked by the Rad50 dimer, although ATP is required for nuclease activities of the complex (42–44).

Here, we further investigate the ATP-dependent nuclease activities of the SbcCD complex. We characterize the influence of topology and length of DNA on stimulating SbcCD’s ATPase, showing that relaxed DNA more efficiently triggers ATP turnover than supercoiled DNA. The presence of DNA ends primarily increases affinity, whereby SbcCD binds ∼25–30 bp. Whereas exonuclease activity requires ATP binding but not hydrolysis, endonuclease activity is robustly stimulated by blocked ends, ATP hydrolysis and an increased AT-content. Together with quantitative estimation of ATP turnover per cleavage and the requirement of SbcD dimer formation and dynamics, the data suggest a model in which repeated ATP hydrolysis by SbcCD near a blocked end generates a melted DNA amenable for cleavage. Most importantly, we find that DNA cleavage on opposing 3′ and 5′ strands, both at the DNA end and at internal sites, are chemically distinct and the phosphodiester is hydrolysed either at the 3′ or at the 5′ side. The chemical signature suggests a different binding polarity of SbcCD at a DNA end compared to facing a protein block and helps sterically unify endo- and exonuclease reactions.

## MATERIALS AND METHODS

### Cloning, protein expression and purification of SbcCD

The genes encoding SbcD and SbcC were cloned into the plasmids pET21b and pET28 (with a modified multiple cloning site), respectively. The gene encoding SbcD was cloned such that the recombinant protein had a C-terminal hexahistidine tag.

Recombinant SbcCD was produced by co-transforming SbcC and SbcD plasmids into *Escherichia coli* BL-21 (DE3) cells. A single colony was picked and grown in LB media to an OD_600_ of 0.6 at 37°C under aerobic conditions. Recombinant protein expression was induced by addition of 0.5 mM IPTG and the cultures were grown overnight at 18°C. Cells were harvested by centrifugation, resuspended in lysis buffer (25 mM Tris pH 7.5, 150 mM NaCl, 10 mM Imidazole and 5 mM β-mercaptoethanol) and disrupted by sonication. The lysate was cleared by centrifugation and applied onto Ni-NTA resin (Qiagen), followed by 2 wash steps with Lysis buffer and subsequent elution (25 mM Tris pH 7.5, 100 mM NaCl, 200 mM Imidazole and 5 mM β-Mercaptoethanol). The elution fractions were applied onto a 1 ml Q HiTrap column (GE Healthcare) and eluted with a linear gradient from 0–100% Buffer A (25 mM Tris pH 7.5, 100 mM NaCl) and Buffer B (25 mM Tris pH 7.5, 1000 mM NaCl). SbcCD eluted as one peak at 30% Buffer B and the peak fractions were pooled, concentrated and further purified by size-exclusion chromatography using a Superose 6 10/30 GL column (GE Healthcare) equilibrated with Buffer C (50 mM Tris pH 7.5, 150 mM NaCl, 10% glycerol). SbcCD eluted as a single peak and the fractions of interest were pooled, concentrated and flash frozen in 10 μl aliquots.

### DNA substrates

For ATPase activation, ΦX174 RFI, RFII or Virion DNA (New England BioLabs®) was used. Linear plasmid DNA was produced by treating ΦX174 RFI with PsiI (New England BioLabs®) followed by heat inactivation.

All oligonucleotides were purchased from Metabion (Planegg, Germany) and purified *via* polyacrylamid gels. RB22 (CGGGTAGTAGATGAGCGCAGGGACACCGAGGTCAAGTACATTACCCTCTCATAGGAGGTG) and RB27 (CACCTCCTATGAGAGGGTAATGTACTTGACCTCGGTGTCCCTGCGCTCATCTACTACCCG) were annealed in annealing buffer (50 mM NaCl, 25 mM Tris pH 7.5, 10 mM MgCl_2_) with a molar excess of 1.1 of unlabeled oligo over the labeled oligo. Oligonucleotides for ATPase and DNA binding assays had a different sequence and were annealed in a 1:1 molar ratio. HS 21 (CGCTTTATCAGAAGCCAGACATTAACGCTTCTGGAGAAACTCAACGAGCTGGACGCGGAT) was annealed to the complement HS37 (ATCCGCGTCCAGCTCGTTGAGTTTCTCCAGAAGCGTTAATGTCTGGCTTCTGATAAAGCG). If shorter double-stranded DNA was used, the HS21 sequence was trimmed on the 3′ end and annealed to the oligonucleotide with the respective complement sequence. For the fluoresecence anisotropy binding experiments, the dsDNA was 6-FAM labeled on the 5′ terminus.

### Nuclease assay

Nuclease assays were carried out in assay buffer (25 mM Tris pH 7.5, 50 mM KCl, 5 mM MgCl_2_, 1 mM MnCl_2_, 0.1 mg/ml BSA, 1 mM DTT) with 1000 nM SbcCD (heterotetramer) and 200 nM of DNA substrate. Where indicated, reactions were supplemented with a 15-fold excess of a single-chain variable fragment against fluorescein (FAM-scFv) (45) or Streptavidin (IBA) over DNA concentration. Unless specified otherwise, reactions were started by DNA addition. Reactions containing DNA with free ends were incubated at 37°C for 15 min, reactions containing end-blocked DNA were incubated for 5 min. Reactions were terminated by mixing 10 μl of the reaction with an equal volume of 2× loading buffer (8 M urea, 20 mM EDTA, 6% Ficoll^®^ 400).

For kinase and phosphatase treatment, the nuclease reactions were terminated by heating to 80°C for 15 min. 10 μl of the nuclease reaction were treated with either T4 Polynucleotide Kinase or Antarctic Phosphatase (New England BioLabs^®^) in the enzyme-specific 1× reaction buffer in a final volume of 20 μl. The reactions were terminated by adding equal volume of 2× loading buffer. To generate short cleavage products, the 60 bp substrate was treated with ExoIII, DnaseI (both New England BioLabs®) and Benzonase^®^ (Merck Millipore) according to manufacturer's specifications.

Reaction products were resolved on 12% denaturing polyacrylamide gels (Rotiphorese^®^ DNA sequencing system) in 1× TBE buffer. Gels were run for 90 min at a constant power of 32 W and scanned by a Typhoon fluorescence imager (GE healthcare). 6-FAM-labeled substrates were imaged with a 473 nm laser and 510 nm filter. The images were analyzed and integrated with the ImageJ software.

### ATP Hydrolysis assays to measure steady-state kinetics

To monitor the hydrolysis rate of ATP, the hydrolysis of ATP was coupled to oxidation of NADH, which can be monitored spectrophotometrically. The reaction buffer contained NADH (0.35 mM), pyruvate kinase/lactate dehydrogenase (20 U/ml PK, 30 U/ml LDH), phosphoenol pyruvate (2 mM) and ATP (1 mM). The assays were conducted at 37°C in assay buffer (25 mM Tris pH 7.5, 50 mM KCl, 5 mM MgCl_2_, 1 mM MnCl_2_, 1 mM ATP, 0.1 mg/ml BSA, 1 mM DTT) and the reaction was started by the addition of SbcCD. The rate of NADH decay/oxidation was monitored fluorometrically by measuring the absorbance at 340 nm on an Infinite M1000 microplate reader (Tecan) at 37°C over a period of 20 min. (46). Estimates of kinetic data (*k*_cat_, *K*_M_, *K*_act_) were determined by fitting reaction data to the Michaelis-Menten equation with Prism (GraphPad).

### Fluorescence anisotropy DNA binding assays

SbcCD dilutions were prepared in assay buffer (25 mM Tris pH 7.5, 50 mM KCl, 5 mM MgCl_2_, 1 mM MnCl_2_, 1 mM DTT) and mixed with the DNA substrate (5 nM final assay concentration, in assay buffer) in a 1:1 (v/v) ratio. After incubation for 20 min at 25°C, the fluorescence anisotropy was measured at an excitation wavelength of 470 nM and emission wavelength of 520 nM. Data were analyzed with Prism (GraphPad) and *K*_D_ values determined by fitting the anisotropy data to a bimolecular equilibrium model: Y$\ = {\rm{\ Af}} - ( {{\rm{Af}} - {\rm{Ab}}} )\frac{{\rm{x}}}{{{\rm{Kd\ }} - {\rm{\ x}}}}$

## RESULTS

### DNA ends and DNA topology differentially stimulate SbcCD’s ATPase

Human and yeast MRN/X have ATPase rates <0.1 ATP/min, which is stimulated 20-fold for human MRN and 10-fold for yeast MRX by linear double-stranded DNA (dsDNA) (47,48). In the absence of DNA, we obtained a similarly low ATPase activity with a *k*_cat_ of 0.008 s^−1^ for SbcCD. 60 base pair (bp) dsDNA stimulated the ATPase hydrolysis 26-fold, while single-stranded DNA (ssDNA) had no effect. SbcCD has a *K*_M_ for ATP hydrolysis of 46 ± 6 μM in the presence of dsDNA (Supplementary Figure S1A), in the same range as human MR(N) and phage T4 gp46/47 (48,49). Altogether, SbcCD displays similar basal ATPase characteristics as its orthologs in other domains of life.

**Figure 1.**
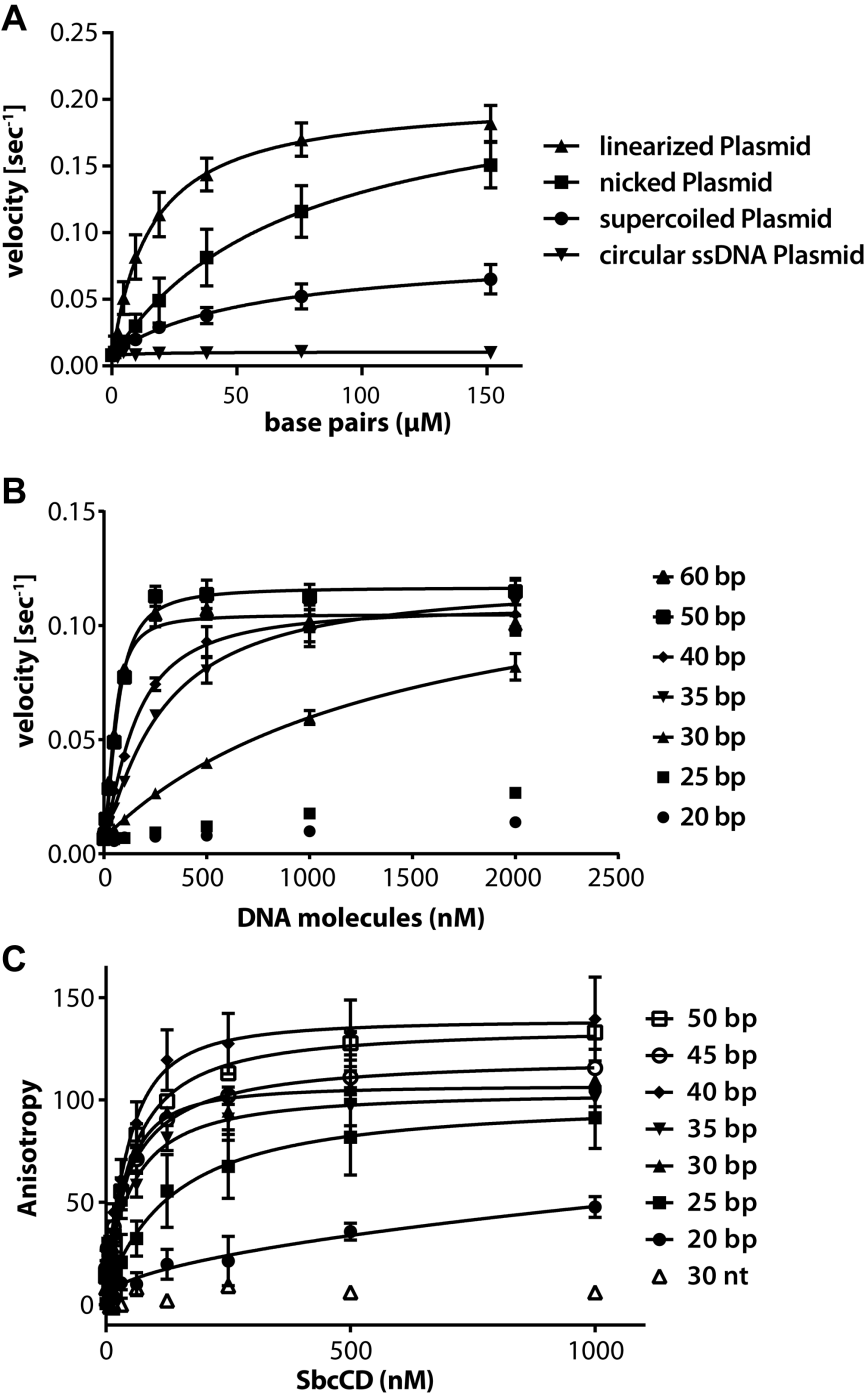
ATP hydrolysis stimulation and DNA binding of the SbcCD^wt^ complex. (**A**) The ATP hydrolysis rate of SbcCD^wt^ was measured in dependence to increasing plasmid DNA concentrations. Bacteriophage ΦX174 Plasmid DNA (5386 bp in length) was added as single-stranded, supercoiled, nicked or linear DNA. The data were fit to a Michaelis–Menten equation, error bars indicate the deviation from three replicates. (**B**) DNA stimulation of ATP hydrolysis by the nuclease-deficient SbcCD^H84Q^ complex. The steady-state ATPase rates were measured at 37°C in the presence of 1 mM ATP, 5 mM MgCl_2_ and 1 mM MnCl_2_. DNA with 20–60 bp in length was added as an activator. The data was fit to a Michaelis-Menten equation, error bars represent the standard deviation of three measurements. (**C**) DNA binding of SbcCD^H84Q^ to 20–50 bp DNA was assayed in the presence of 1 mM ATP, 5 mM MgCl_2_ and 1 mM MnCl_2_. DNA concentration was kept at 5 nM; the SbcCD^H84Q^ concentration ranged from 2 to 1000 nM. Data points represent the change in fluorescence anisotropy and the data were fit to a 1 to 1 binding equation. Error bars represent the deviation from three independent experiments.

To test to the role of DNA topology on ATP hydrolysis we measured the stimulatory effect of a 5.4 kb plasmid in (i) supercoiled (ii) relaxed (iii) linearized and (iv) single-stranded state. SbcCD and ATP were kept at constant concentrations and the DNA was added in increasing amounts. Circular single-stranded DNA did not stimulate ATP hydrolysis of SbcCD. The supercoiled plasmid activated the ATPase up to 9-fold (*k*_cat_ = 0.072 ± 0.006 s^−1^), whereas the nicked plasmid activated the ATPase 26-fold (*k*_cat_ = 0.21 ± 0.02 s^−1^). The increased activation of relaxed DNA is clearly direct and not attributable to higher affinity binding, since the *K*_act_ (concentration at half maximal activation) is 2-fold lower for the supercoiled plasmid than for the nicked plasmid. It should be noted that even though nicked and supercoiled DNA can stimulate SbcCDs ATPase activity, we did not observe any DNA cleavage activity. In contrast, linearized plasmid DNA is readily degraded (Figure 1A, Supplementary Figure S2). Comparing the ATPase stimulation of a 60 bp dsDNA and a linearized plasmid revealed that 60 bp dsDNA is able to stimulate the ATPase activity stronger than the linearized Plasmid at the same molarity (Supplementary Figure S3).

Linearizing the plasmid with PsiI, which generates blunt ends, stimulated *k*_cat_ of ATP hydrolysis (0.200 ± 0.007 s^−1^) similar to a nicked plasmid, however *K*_act_ is lowered 4-fold, showing an increased affinity at lower DNA concentrations. One DNA break/hairpin in an *E. coli* cell would correspond to an approximately nanomolar concentration of DNA breaks, not taking into account molecular crowding effects (50). At this concentration, the ATPase was activated 7-fold by linear DNA, but only 2-fold by nicked DNA (Figure 1A, Supplementary Table S1C).

Our data show that both topological features of the DNA and the presence of DNA ends affect the ATPase rate of SbcCD. *k*_cat_ is higher with relaxed DNA than with supercoiled DNA, but it is not further enhanced by the presence of DNA ends. DNA ends appear to primarily increase the affinity of the complex but not its intrinsic ATP turnover rate.

### DNA length requirements for DNA binding and ATPase activity

To determine the minimal length that is required for robust ATPase activation we tested dsDNA from 20 to 60 bp in length. A nuclease-deficient mutant (SbcCD^H84Q^) of SbcCD was used in the assays to prevent DNA degradation during the course of the study. H84Q decreased the ATPase activity of SbcCD by ∼15% (Supplementary Figure S1A). 20 and 25 bp DNA did not substantially stimulate ATP hydrolysis of SbcCD. A moderate activation was obtained with 30 bp DNA. Increasing the length of DNA to 35 bp and longer robustly stimulated the ATPase rate (Figure 1B). Since we used concentrations of the SbcCD heterotetramer of 500 nM to also probe for effects of shorter DNAs, binding affinities well <500 nM of DNA >40 bp (see below) did not allow us to derive affinities in these studies. Rather, we titrated DNA end binding by SbcCD. In the case of 50 and 60 bp DNA, the near maximal ATPase activation was already obtained at a concentration of 250 nM DNA, where the concentration of DNA ends is the same as the concentration of SbcCD. With shorter DNA, steric competition might prevent productive (ATPase) binding of two complexes to both DNA ends. In any case, the 20–25 bp minimal requirement for the DNA stimulation of ATPase activity coincides well with the footprint of ATP bound NBD dimers of Rad50, which is ∼20 bp.

To see how the DNA length dependent activation of the ATPase coincides with DNA binding affinity, we measured DNA interaction through changes in the fluorescence anisotropy of labeled DNA of different lengths. We first tested DNA binding in the presence and absence of ATP. SbcCD did not bind single stranded DNA (ssDNA) in either the presence or absence of ATP. In addition, we could not detect binding to dsDNA in the absence of ATP, indicating that the formation of engaged NBDs is critical for DNA binding by SbcCD (Figure 1B, Supplementary Figure S1C). Next, we evaluated the affinity of SbcCD in the presence of 1 mM ATP for dsDNA oligonucleotides, ranging from 20 to 50 bp in 5 bp increments. SbcCD shows low affinity to 20 bp DNA and moderate affinity to 25 bp DNA (*K*_D_ = 146 ± 46 nM). However, lengthening the DNA to 30 bp DNA resulted in a notable increase in binding affinity (*K*_D_ = 43 ± 7 nM). Further lengthening of the DNA did not affect the *K*_D_, which remained in the range of 50 to 60 nM (Figure 1C, Supplementary Figure S4). Therefore, maximal DNA end binding has a ‘footprint’ of ∼25–30 bp, whereby affinity is not enhanced by longer DNA.

### Characterizing the nuclease activities of SbcCD on 60 bp DNA

MRN/X and SbcCD comprise nuclease activities that are conserved amongst bacteria, yeast and human, but also show unexplained differences. Conserved functions are the (i) 3′-5′ exonuclease on dsDNA, (ii) cleavage of dsDNA adjacent to a protein-blocked DNA end, (iii) cleavage of hairpin structures on the 5′ side of a hairpin, and (iv) cleavage of 3′ and 5′ overhangs (7,11,12,25). On a long hairpin substrate, SbcCD displayed a progressive ‘binary’ endonuclease that nicks both DNA strands and introduces DNA double-strand breaks in 10 bp intervals (32), an activity that has not been reported so far for enzymes from other species.

**Figure 2.**
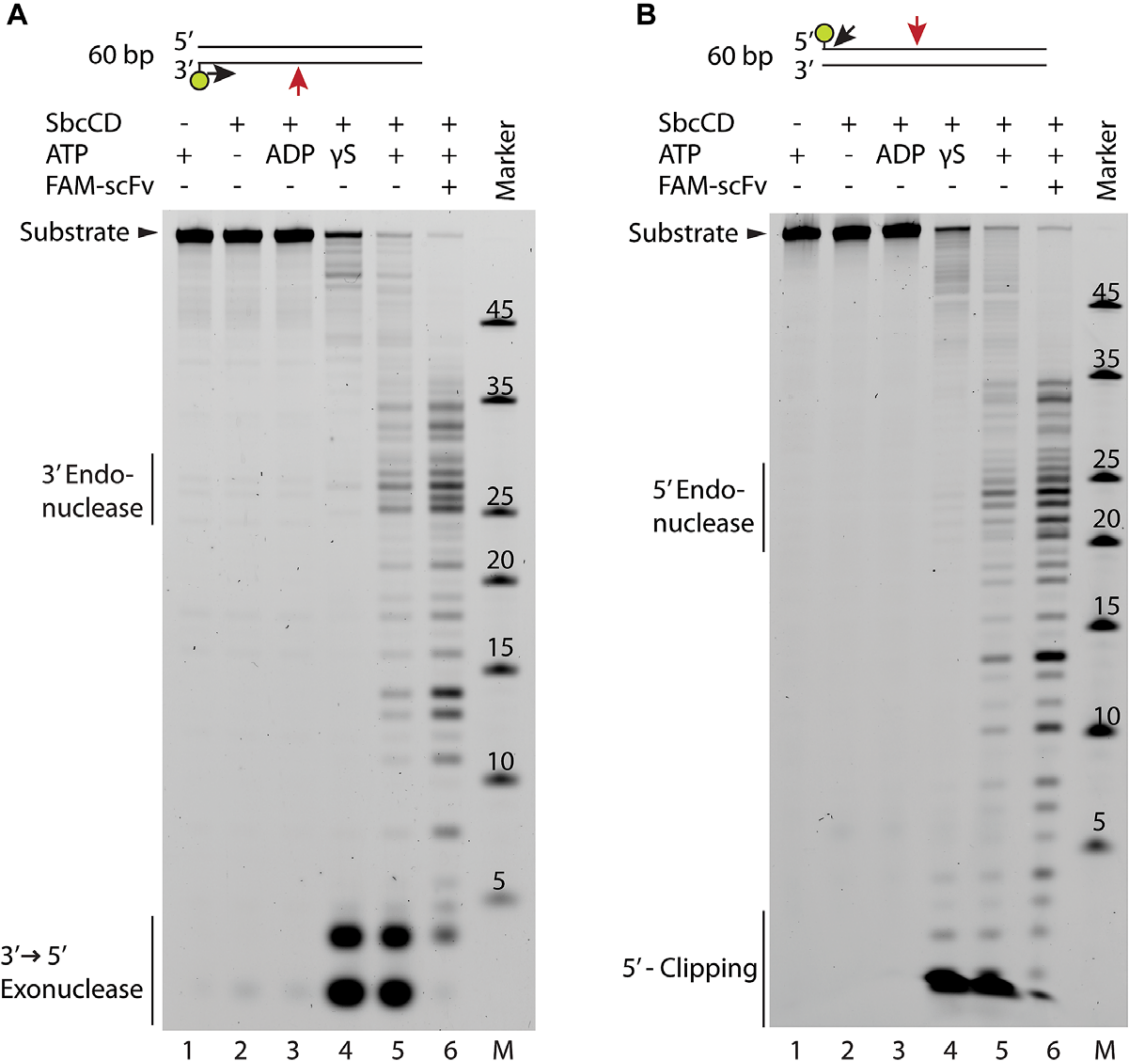
Nuclease activity of SbcCD^wt^ towards 60 bp DNA. (**A**) SbcCD^wt^ was assayed in the presence of 1 mM ADP or ATP(γS), 5 mM MgCl_2_ and 1 mM MnCl_2_ at 37°C. The 60 bp DNA substrate was labeled on the 3′ end with 6-FAM. FAM-scFv is a single chain fragment that binds to Fluorescein dyes with a *K*_D_ of 4 nM. Reactions with unblocked DNA substrates were quenched after 15 min, reactions containing FAM-scFv were quenched after 5 min. The cleavage products were separated by Urea-PAGE and visualized on a Typhoon scanner. Major cleavage products of SbcCD are depicted above. (**B**) Nuclease assay as in (A), but with 5′ labeled 60 bp DNA.

To further characterize the nuclease activities of SbcCD, we tested the degradation of a 60 bp DNA labeled with a fluorescent dye at the 5′ or 3′ terminus. In the absence of ATP and presence of ADP, faint low molecular weight products appear which may point to a residual 3′ exonuclease activity independent of ATP. The presence of non-hydrolysable ATPγS induces robust 3′-5′ exonuclease activity of SbcCD (Figure 2A, lanes 2–4). Of note, we also observed a clipping activity near the 5′ end of DNA (Figure 2B, lanes 4 and 5). This is likely due to prior degradation of the 3′ terminus, as phosphorothioate protection of the complementary 3′ end led to a high reduction of this activity (Supplementary Figure S5, lanes 5–8). In the presence of ATP, internal DNA cleavage products could be detected. They appear most prominently at 27 bp from the 3′ end and 23 bp from the 5′ end (Figure 2A and B, lane 5). We then tested the cutting efficiency in the presence of a protein block, formed by an anti-fluorescein antibody derived single-chain fragment variable (FAM-scFv) (45). The block is similar in size to the streptavidin–biotin conjugate used in previous experiments (7) and mimics a blocked DNA end or a DNA–protein crosslink (DPC), structures that often occur at DNA double-strand breaks (DSBs). The presence of a protein block stimulated the endonucleolytic cleavage, as predominantly endonuclease products appeared (Figure 2A and B, lane 6). In contrast to the exonuclease that is fully active in the presence of ATPγS, the block-stimulated endonucleolytic incision is highly decreased in presence of ATPγS and therefore promoted by ATP hydrolysis (Supplementary Figures S6 and S7A, lanes 4 and 5). An 80 bp duplex DNA was also incised 27 bp from the DNA end, therefore the 27 bp distance was determined by the labeled DNA end (Supplementary Figure S5, lanes 1–4).

### SbcCD’s endonuclease activity is sensitive to the melting stability of DNA

The mechanism how Mre11 family proteins such as SbcD incise double-stranded DNA is not known yet. Structures of Mre11 with dsDNA reveal that the manganese ions of Mre11 are concealed in the active site and the bound B-DNA is at least 5 Å away from a position that could be productive for cleavage. At least endonucleolytic activity would require, from sterical considerations on the basis of available structures, DNA duplex unwinding in order to reach the active site metals. Indeed unwinding activity was reported for the human MRN. This process was Nbs1- and ATP-dependent and enhanced by a 44 nucleotide overhang (11,51). Processive DNA unwinding of a 50 bp duplex was also reported for MR from *Thermotoga maritima* (42).

To relate putative melting of duplex DNA to nucleolytic incision, we designed DNA substrates with different local AT and GC contents, since the local stability of B-DNA can be tuned via the GC/AT content (52). 60 bp DNA was modified from position 15 to 29 (relative to the 6-FAM dye) with, (i) mixed AT/GC-content, (ii) 100% AT-content or (iii) 100% GC-content. The endonuclease activity was tested in both the presence and absence of a protein-bound DNA end. The unlabelled DNA end was protected from degradation by phosphorothioates on the 3′ terminus.

**Figure 3.**
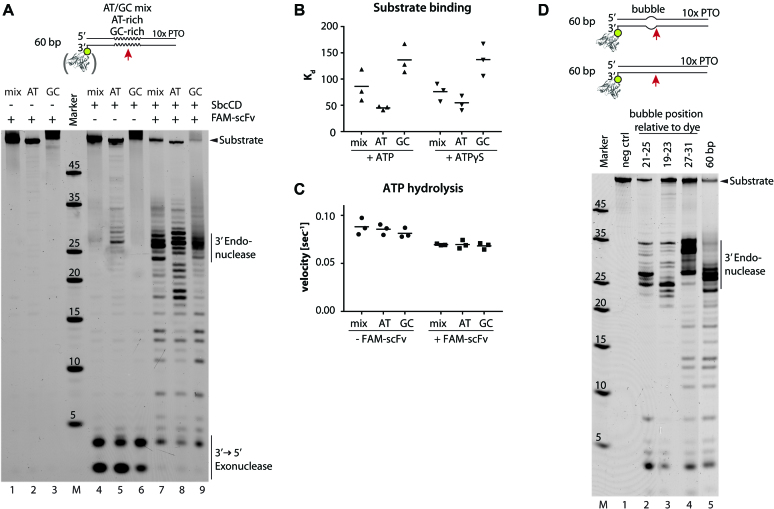
SbcCD cleaves double-stranded DNA dependent on the local AT/GC concentration and pre-melted DNA stretches. (**A**) Nuclease activity of SbcCD^wt^ was assayed in the presence 1 mM ATP, 5 mM MgCl_2_ and 1 mM MnCl_2_ at 37°C. The DNA was labeled on the 3′ end with 6-FAM and contained different AT/GC concentrations from position 15 - 29 (relative to the dye). FAM-scFv - single chain fragment that binds to the 6-FAM dye. (**B**) Dissociation constants (*K*_D_) of SbcCD^H84Q^ were obtained by fitting fluorescence anisotropy data to a 1 to 1 binding equation. Bar represents the mean of three values. (**C**) The steady-state ATP hydrolysis rates of SbcCD^wt^ were measured during the nuclease reactions in (A). Bar represents the mean of three values. (**D**) The nuclease activity of SbcCD^wt^ towards 60 bp DNA with pre-melted DNA regions (bubble) was tested. SbcCD^wt^ cleaves the DNA substrates 5′ to the pre-melted region

As observed in previous assays, SbcCD cleaved 60 bp DNA with mixed AT/GC content 27 bp from the 3′ end with moderate activity (Figure 3A lane 5). Endonuclease became more efficient with AT-rich DNA and almost vanished with GC-rich DNA (Figure 3A lanes 6–7). Therefore, SbcCD’s endonuclease is sensitive to local stability of the dsDNA and, as judged from AT/GC content, performs better when the DNA can be melted more easily. Of note, the cutting preference at AT-rich regions was overridden by a protein block and the three duplex DNAs were incised with apparently similar efficiencies (Figure 3A, lanes 8–10).

We next interrogated whether DNA stability affects ATPase rates or DNA affinity. Indeed, SbcCD bound AT-rich DNA with a higher affinity than GC-rich DNA. The affinity increased 3-fold from a *K*_D_ of 132 nM to 45 nM. In contrast, the AT/GC content did not affect the ATPase activation under the nuclease assay conditions (Figure 3B and C). Thus, the increase in endonucleolytic efficiency or increase in binding affinity to AT-rich DNA cannot be attributed to higher ATPase rates. It rather appears that continuous ATPase activity generates perhaps melted or otherwise conformationally altered DNA that is more efficiently bound and cleaved by the nuclease.

To see how efficiently ATP hydrolysis and endonuclease are coupled, we monitored the rate of ATP hydrolysis under conditions identical to the nuclease assay (Figure 3C). In the course of 60 bp DNA degradation, SbcCD hydrolyzed approximately 800 molecules ATP to degrade one molecule of DNA in the absence of a protein block. Protein-blocked DNA used up 200 ATP molecules per DNA cleavage. Since exonuclease activity is observed in the presence of the non- or slowly hydrolysable analog ATPγS, it appears that cleavage of terminal nucleotides does not strictly require rounds of ATP hydrolysis. However, the lack of endonuclease activity observed in the presence of ATPγS, and consistently the high numbers of ATP hydrolysis events per endonucleolytic cleavage suggest that rounds of ATP hydrolysis catalyze an inefficient or reversible step prior or during cleavage.

Encouraged by these novel findings, we designed DNAs with unpaired stretches of five nucleotides (bubbles) mimicking melted DNA at various distances from the DNA ends. A protein-blocked 60 bp fully base paired duplex was digested in the previously characterized pattern, having a major incision species at 27 bp. Introduction of the bubble from position 27 to 31 leads to incision events at position 31–35, another prominent cleavage product appeared at 27 bp. Locating the bubble at position 19–23 leads to a major cleavage site at 25 nucleotides, position 21–25 guides the incision to 27 bp (Figure 3D, lanes 2–5). Therefore, SbcCD cleavage occurs at the 5′ side of unpaired DNA. Increasing the length of the bubble to seven nucleotides reduced cutting efficiency, so it is unlikely that SbcCD unwinds DNA very extensively at this site. In the presence of non-hydrolysable ATPγS we could not detect endonucleolytic degradation with any of the substrates (Supplementary Figures S7 and S8). These experiments suggest that the endonucleolytic incision of duplex DNA by SbcCD is sensitive to the thermodynamic stability of B-DNA and that some local changes in DNA structure occur prior to endonuclease activity as a result of ATP hydrolysis cycles. However, preformed bubbles do not relieve the necessity of ATP hydrolysis and are also poorer endonuclease substrates than base-paired DNA. For instance, ATP hydrolysis could also help load the complex onto internal DNA in the presence of a block, but other scenarios are also possible.

### SbcD dimer interface dynamics during endonucleolytic degradation of SbcCD

**Figure 4.**
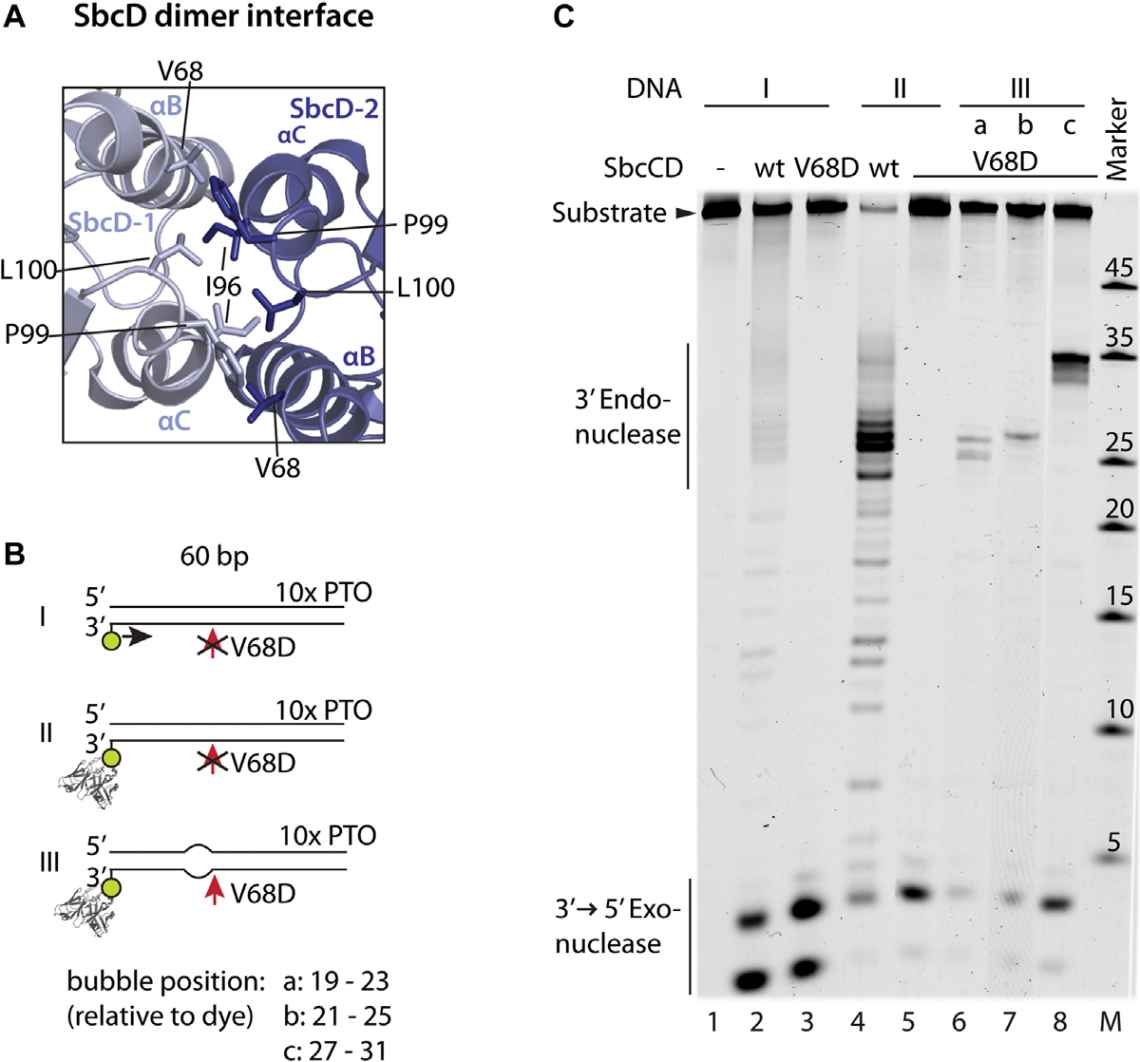
SbcD dimer disruption affects endo- but not exonuclease activity. (**A**) SbcD homodimeric interface (PDB: 4M0V). The view shows the SbcD-SbcD four-helix bundle interaction. The interaction is mainly mediated by a hydrophobic cluster consisting of Val68, Iso96, Phe99, and Leu100 on the top of the four-helix bundle. (**B**) DNA substrates are schematized that were used in (C). (**C**) Nuclease activities of SbcCD^V68D^ was assayed in the presence 1 mM ATP, 5 mM MgCl_2_ and 1 mM MnCl_2_ at 37°C. SbcCD^V68D^ retains exonuclease activity, but looses endonuclease activity on duplex DNA. The presence of bubbles re-establishes the endonuclease activity of SbcCD^V68D^.

To investigate the impact of the SbcD dimer state during nucleolytic processing, we compared the nuclease activities of SbcCD^wt^ to a SbcCD complex with a destabilized SbcD interface. The dimeric assembly of two SbcD protomers is mediated by a conserved four-helix bundle interaction which bears a hydrophobic cluster consisting of Val68, Ile96, Phe99 and Leu100 (Figure 4A) (53). The interface was weakened by mutating Val68 to a negatively-charged aspartate (SbcD^V68D^).

Right-Angle Light Scattering (RALS) analysis of the SbcD nuclease and capping domain showed that SbcD mainly forms a monomer during size-exclusion chromatography in the absence of SbcC, with a very slight residual fraction of 4% dimeric SbcD. This residual dimer is absent in SbcD^V68D^. Therefore, SbcD itself appears to be an at least transient homodimer whereas the addition of a second interface by SbcC in the presence of ATP induces stable complex formation of the SbcCD – head domain and the full length complex (Supplementary Figures S9A and B and S10). Mutations at similar sites in yeast and bacteriophage Mre11 have been analyzed before and found to impact on the viability *in vivo* or nuclease characteristics *in vitro* (35,54).

SbcCD^V68D^ degrades dsDNA exonucleolytically in an ATP-dependent fashion similar to SbcCD^wt^. Therefore, the three-dimensional fold of SbcD and the catalytic site is functional and the association between DNA and SbcCD^V68D^ is intact. Furthermore, a fully functional SbcD dimer interface is apparently not required for exonuclease activity, similar to the dispensability of ATP hydrolysis (but not ATP binding) for the exonuclease. However, SbcCD^V68D^ did not show significant endonucleolytic activity and we could not detect any dsDNA degradation in the presence of ATPγS (Supplementary Figure S8). Introduction of a bubble structure re-established the endonuclease activity. The positions of the incision were identical to SbcCD^wt^, however, the cutting efficiency reduced by approximately 5-fold, depending on the position of the bubble (Figure 4C). Therefore, destabilization of the dimerization interface of SbcD correlated with the loss of endonuclease activity of SbcCD. The presence of unpaired DNA re-established the ATP dependent endonuclease activity to some extent.

The nuclease and capping domain of SbcD^V68D^ display similar ssDNA endonuclease activity towards a covalently closed single-stranded plasmid DNA as SbcD^wt^. Full-length SbcCD^wt^ and SbcCD^V68D^ required ATP for ssDNA cleavage. Both proteins cleaved ssDNA with a similar efficiency in the presence of ATP. SbcCD^wt^ also cleaved in the presence of ATPγS, whereas the endonuclease of SbcCD^V68D^ was strongly reduced (Supplementary Figure S11A and B).

These data suggest that a destabilized SbcD interface induces a defect in SbcCD that affects the endo- but not the exonuclease activity. SbcCD^V68D^ also cleaves single-stranded plasmid DNA and bubble structures in the presence of ATP. Therefore, the defect could be a dysfunction of SbcCD^V68D^ to generate a DNA substrate which is competent for endonucleolytic cleavage. Introduction of a pre-melted stretch compensates this defect and SbcCD^V68D^ regains endonuclease activity. The most likely explanation is that SbcD dimers with a fully functional interface are needed to generate melted DNA.

### SbcCD cleaves opposing strands of dsDNA with different chemistries, leaving 3′ and 5′ phosphates respectively

To further investigate and characterize the mode of cleavage catalysis, we treated the cleavage products with Antarctic Phosphatase and T4 Polynucleotide Kinase enzymes and compared them to the untreated cleavage products. The addition or removal of the negatively-charged phosphate changed the mobility of the oligonucleotides during electrophoresis and allowed us to detect the presence or absence of a phosphate group at the unlabeled termini.

**Figure 5.**
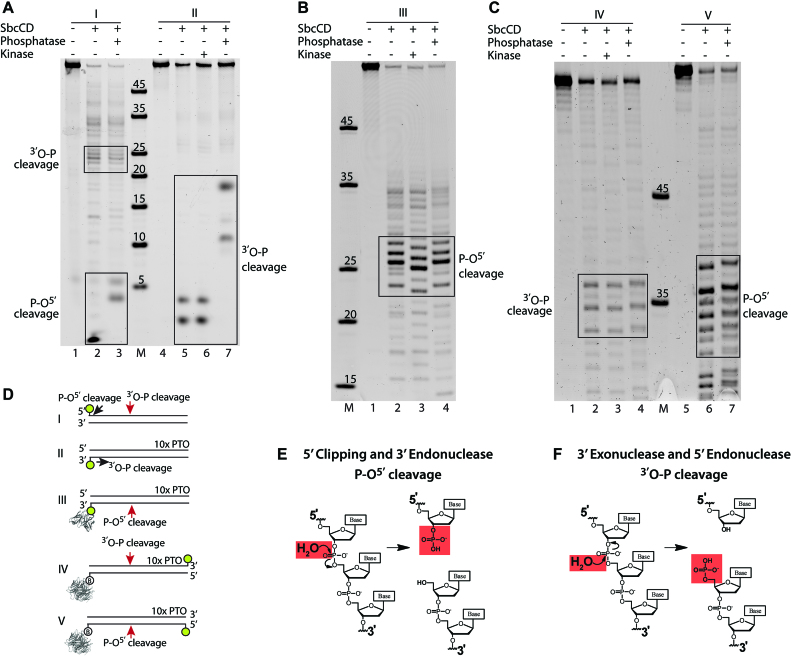
The position of SbcCD’s DNA backbone cleavage is cleavage- and strand-specific. (**A**) SbcCD^wt^ was assayed in the presence of 1 mM ATP, 1 mM MnCl_2_ and 5 mM MgCl_2_ at 37°C. The cleavage products of the quenched nuclease reactions were treated with T4 Polynucleotide Kinase or Antarctic Phosphatase to remove or add a phosphate to the DNA ends. The altered electrophoretic migration indicates the absence or presence of a phosphate of the cleavage products. (**B**) Nuclease assay as in (A), but with 60 bp DNA which was protein bound DNA end inducing endonuclease activity. (**C**) Nuclease assay as in (A), but oligos were labeled at the unblocked DNA end. (**D**) DNA substrates and respective cleavage products in (A–C) are schematized. (**E** and **F**) The chemical drawing shows the S_N_2 reaction during exo- and endonucleolytic cleavage of the phosphate backbone.

Analysis of a 60mer DNA duplex with a 3′ label revealed that the 3′ exonuclease products were not shifted by kinase treatment but by phosphatase treatment, indicating the presence of a 5′ phosphate and cleavage of the P–O^5′^ phosphoester bond (Figure 5A, lanes 6–8). The resulting cleavage products were validated using well characterized nucleases ExoIII, DNase I and Benzoase (Supplementary Figure S12, 55–58). Comparison of the cleavage products produced by the different nucleases suggests that SbcCD can direcly cleave the phosphodiester linkage of the 6-FAM at the 3′ end. The major cleavage at the DNA end was observed to be between base 3 and 4 (from the 3′ end), liberating a trinucleotide (Supplementary Figure S12A). We also see to a smaller amount cleavage between bases 1–2, 2–3, 4–5 and 5–6, so there is apparently some structural flexibility in recognition of 3′ 6-FAM bound DNA ends by SbcCD. This is consistent with the ability of SbcCD to recognize and cleave hairpins (33). Comparison of the exonuclease cleavage polarity of SbcCD^wt^ and SbcCD^V68D^ did not indicate any differences (Supplementary Figure S13).

Analysis of the same 60mer DNA duplex, but with a 5′ label revealed two sets of products; endonuclease products between 20 and 25 bp from the 5′ end, and short 5′ clipping products. The endonuclease cleavage products were not shifted by phosphatase treatment and therefore contain a free 3′-OH, similar to the exonuclease cleavage products on the complementary DNA strand. Interestingly, the 5′ clipping products were shifted by phosphatase treatment, indicating the presence of a 3′-phosphate and cleavage of the ^3′^O–P bond. Therefore, the 5′ clipping apparently has a different chemistry to the 3′-5′ exonuclease and 5′ endonuclease activities (Figure 5A, lanes 1–3).

Next, we assessed the cleavage chemistry of the 3′ endonuclease activity, using a protein block at the 3′ label. Endonuclease products between 23–27 bp in length were not affected by phosphatase treatment, but migrated faster after kinase treatment. Thus, these products contained a 5′-OH, indicating cleavage of the ^3′^O–P bond (Figure 5B). This is similar to the 5′ clipping reaction, but apparently different to the endonucleolytic cleavage of the 5′ strand. However, the 5′ endonuclease produced cleavage fragments harbour a 3′-OH, similar to the 3′-5′ exonuclease.

The observed 3′-OH could either be directly produced from the endonuclease, or alternatively result from a 3′-5′ exonuclease reaction of the nicked DNA. To clearly attribute the observed cleavage chemistries to endonuclease reactions, we analyzed DNA substrates that were fluorescently labeled on the DNA termini opposite to the protein-blocked DNA end. Looking at the endonucleolytic products around 35 bp from the labeled end (∼25 bp from the blocked end), we observed that the kinase treatment did not result in a different mobility, whereas phosphatase treatment led to slower migration of both 3′ and 5′ labeled DNA (Figure 5C). These data suggest that—looking from the blocked DNA-end—the 3′ strand is cleaved at the P-O^5′^-bond, leaving a 3′-phosphate, whereas the 5′ strand is cleaved at the ^3′^O-P-bond, leaving a 5′-phosphate (Figure 5E and F). It is likely that some cleavage products that do not shift upon kinase treatment originate from a consecutive endo–exo activity as has been observed for the human MRN complex (33).

In summary, we unexpectedly observe that SbcCD cleaves opposing strands with a chemically different nuclease reaction. The exonuclease and clipping activities at the DNA end operate in such a way that the phosphate groups stay on the short nuclease products, leaving an unphosphorylated DNA end with free 3′-OH and 5′-OH. The DNA end proximal endonuclease activities show the reverse polarity, as if the SbcCD complex is geometrically flipped and just operates in the other direction. Here, both phosphate groups stay at the newly formed (unblocked) DNA end, while the dsDNA portion that contains the block contains a 3′-OH and 5′-OH.

## DISCUSSION

Orthologs of the Mre11–Rad50 complex function in the processing of terminal DNA structures in all kingdoms of life. While the primary substrates and targets for the ATP-regulated nuclease activity, i.e. arrested topoisomerase–DNA complexes, hairpins, abnormal replication intermediates and blocked DNA ends differ in different species, Mre11–Rad50 complexes share similar nuclease activities: they possess hairpin opening and 3′-5′ dsDNA exonuclease activities and can cleave blocked DNA ends *via* an endonuclease activity ∼15–25 bp inward from the block (4,5,12,25,33). Collectively, these activities have been suggested to clear diverse types of blocked ends and prepare them for recombinational repair. A common mechanistic basis for these nuclease activities by Mre11/SbcD and their regulation or activation by ATP binding and hydrolysis by Rad50/SbcC remains to be established.

In SbcCD, DNA binding, nuclease activation and ATP binding or turnover are strongly coupled. In the absence of ATP, we neither observe DNA binding nor detectable nuclease activity. Robust activation of SbcCD’s ATPase by DNA, or DNA binding to SbcCD requires 25–30 bp, by and large consistent with a structural footprint of ∼20 bp DNA on the ATP-bound NBD dimer of Rad50/SbcC proteins (41,42). The slightly longer DNA needed for full affinity binding and especially for full stimulation of ATP hydrolysis by SbcCD could indicate either a different conformation of the complex when bound to a DNA end, or reflect some cooperative effects when more than one complex is bound, as for instance indicated by recent studies (32).

Interestingly, we find that the ATPase is most robustly activated by relaxed DNA, either in circular or in linear form, but much less by supercoiled DNA. The presence of DNA ends does not increase the maximum ATP turnover rates, but rather increases the affinity of the DNA for SbcCD and thus activates the ATPase at much lower DNA concentrations. Structural results showing that Rad50/SbcC’s NBDs do not directly recognize DNA ends is consistent with circular DNA being able to fully activate SbcC’s ATPase (42,43,59). Recent single-molecule studies showed that the Mre11 subunit of the MRN complex is necessary for DNA end recognition but not binding to internal DNA (24). It is therefore plausible that high-affinity end-recognition proceeds *via* the SbcD subunit, whereas SbcC’s ATPase is activated by the flanking DNA. However, it is yet unclear how Mre11/SbcD and Rad50/SbcC would cooperate in DNA binding and processing, since current structural studies have not shown that or how both subunits can simultaneously bind to DNA. Of note, the activation of SbcC’s ATPase by relaxed DNA could be a medium-range signal to sense the presence of DNA breaks or palindromes. While former readily leads to loss of superhelical tension, latter can fold into cruciforms and can also lead to local relaxation of DNA in otherwise supercoiled chromosomal DNA.

Even under conditions of full ATP turnover, we do not observe cleavage of circular supercoiled or circular nicked DNA. In contrast, linear DNA is readily degraded. Clearly, the activation of ATP hydrolysis by DNA is not sufficient to trigger endonuclease activity and requires the presence of DNA ends. In the presence of DNA ends, however, ATPγS is sufficient to activate the exonuclease. Equivalent results have been observed previously for SbcCD and the homologous bacteriophage T4 gp46/47 complex, where it was found that cleavage of the terminal 3′ base does not need ATP hydrolysis, but further processive 3′-5′ exonuclease activity does (49). On the contrary, the endonuclease of SbcCD, which is robustly activated by DNA end blocks, requires ATP hydrolysis in our hands, similar to observations made for eukaryotic homologs (5,33,60). Quantification yields a relation of ATP turnover to endonuclease cuts of around 100:1. Likely, a transient, reversible step is necessary for a more infrequent endonucleolytic cleavage. A putative mechanism is that ATP binding by SbcC leads to the transient, but reversible local melting of DNA, which could then be cleaved by SbcD. In support of this hypothesis are our observations with DNA substrates containing base bias (AT-rich and GC-rich), showing an inverse correlation between thermodynamic stability and efficiency of endonucleolytic cleavage. Of note, in other investigations of SbcCD’s nuclease, ATPγS is sufficient to trigger endonucleolytic cleavage (7,32). Perhaps on different DNA substrates or under different experimental conditions, the efficiency of the complex is substantially increased that binding of ATPγS alone is sufficient to prepare DNA for cleavage, relaxing the requirement for the turnover of multiple molecules of ATP. Nevertheless, our findings on the requirement of ATP *vs* ATPγS are generally consistent with what has been observed for the activity of homologs (5,33).

The putative DNA melting required for endonucleolytic cleavage requires a properly formed SbcD dimer, since mutation of the dimer interface reduced endonuclease, but did not reduce exonuclease or endonuclease on a substrate containing a DNA bubble. Structural studies on Mre11 dimers bound to DNA are so far consistent with endonucleolytic cleavage requiring some DNA melting in order for the phosphoesters to reach the active site metals. In addition, the unwinding or melting of short dsDNA has been observed for eukaryotic and bacterial homologs in the presence of ATP (11,42,51). At the DNA end, fraying or other sterical features might already be sufficient for exonuclease activity, requiring only ATPγS-mediated binding of SbcCD to DNA. In the case of internal DNA, however, multiple ATP turnover cycles might be required to open and maintain the DNA in a melted state until it is cleaved by SbcD.

For the endonuclease activity, it was first shown for the *E. coli* homolog SbcCD and subsequently for the eukaryotic MRN and MRX complexes, that Mre11–Rad50 complexes can indeed cleave both strands of the DNA, introducing a DNA double-strand break near the block. To better understand a common or distinct mechanistic nature of these inherent endo- and exonuclease activities of Mre11–Rad50 complex proteins, we analyzed SbcCD’s ATP-dependent nuclease activity in the processing of accessible and inaccessible (protein blocked) DNA ends. We unexpectedly found that the endonucleolytic cleavage reactions at opposing DNA strands are chemically distinct. One strand is cleaved such that the phosphate remains at the 5′O, while the opposing strand retains the phosphate at the 3′O. Using the conventional 5′-3′ directionality of DNA, we refer to former activity as ^3′^O–P cleavage (phosphate remains at 5′O) and to latter as P–O^5′^ cleavage (phosphate remains at 3′O). It was found before that SbcCD cleaves a hairpin at the 5′ side with an ^3′^O–P cleavage (61).

**Figure 6.**
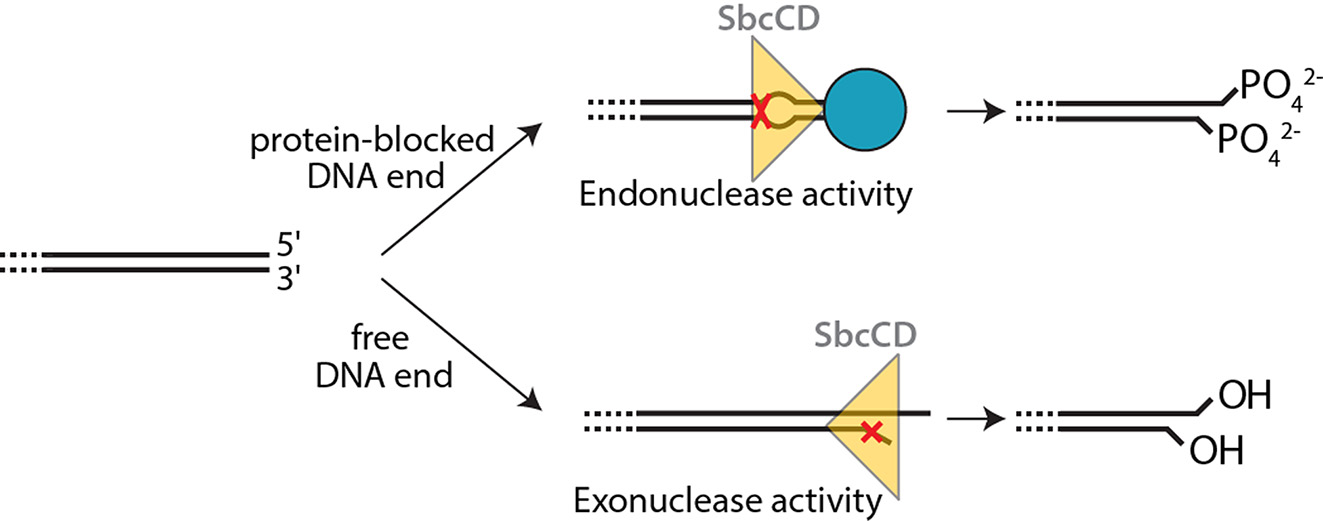
Proposed model for the SbcCD Endo- and Exonuclease mechanism. The SbcCD dimer is shown schematically as a yellow triangle and the cleavage site on DNA is indicated by a red cross. A protein block at the DNA end (blue sphere) stimulates SbcCD’s endonuclease activity. Internal cleavage of the DNA requires ATP hydrolysis, the DNA is melted and a transient bubble is formed. The DNA end is phosphorylated at the 5′ and the 3′ termini (upper panel). For exonuclease activity the SbcD dimer is in a reversed orientation, cleavage could involve fraying at free DNA ends. The DNA end is hydroxylated at the 3′ and the 5′ termini (lower panel).

Interestingly, we also observed these two chemically distinct mechanisms at the cleavage reactions at the DNA end, but with a reversed polarity: in addition to the well-characterized 5′-3′ exonuclease activity (O–P cleavage), we found that SbcCD possesses a 5′ clipping activity with P–O cleavage. The observed pattern of ^3′^O–P versus P–O^5′^ is therefore not determined by the type of reaction (endo- or exonuclease), but sterically determined by the DNA end. In our opinion the most rational explanation is that SbcCD complexes are situated at an accessible DNA end, and at an internal site next to a blocked end with a reversed polarity. A plausible scenario is shown in Figure 6. Here, a SbcD dimer is situated in such a way that the 3′ strand is exonucleolytically clipped with an ^3′^O–P cleavage reaction. If the complex encounters a block through e.g. internal diffusion or scanning, the SbcD dimer could have a different orientation, thus the observed endonucleolytic cleavage at the 5′ strand has the ^3′^O–P cleavage chemistry. Consistent with such a model are recent observations on the basis of the bipolar nuclease reaction: here it was proposed that the hairpin/DNA end-bound terminal SbcCD complex and subsequent SbcCD complexes are biochemically distinct (32). For instance, the terminal complex can perform the hairpin opening reaction but may also temporarily act as a block to stimulate endonuclease reactions by further SbcCD complexes at internal sites. The nature of the 10 bp periodicity of the subsequent endonuclease reactions compared to the 20–25 bp of the initial cleavage from the end is not clear yet and requires further investigation.

How can SbcD catalyze both ^3′^O–P and P–O^5′^ cleavage events? On the basis of its homology to other phosphodiesterases, the di-metal center of Mre11/SbcD is suggested to coordinate and activate the phosphate and attacking hydroxyl ion. Mre11’s nucleolytic cleavage is proposed to be an S_N_2-reaction, which requires the in-line nucleophilic attack of a water molecule or hydroxyl ion (34). To produce cleavage products with different cleavage chemistries, either the positions of the water molecule and the phosphate on the di-metal cluster are reversed or, perhaps more likely, water and phosphate occupy the same position, but the strand polarity is reversed. The currently available crystal structures of archaeal Mre11 homologs bound to DNA have not revealed how DNA coordinates the active site metals, since both strands are equally 10–15 Å away from the two metal ions (35,36). While these structures support the sterical need for DNA melting or deformation of DNA to allow the backbones to reach the active site metals for endonucleolytic cleavage, it is possible that either strand could reach the active site while maintaining the overall direction of the DNA. In such a model, the different strand polarity, together with a maintained water and phosphate coordination at the di-metal center, would result in the observed P–O^5′^ and ^3′^O–P cleavage reactions on opposing strands.

In summary, we deconvolute and unify key aspects of endo- and exonuclease type reactions in SbcCD. We reveal an unexpected chemical asymmetry in the mode of cleavage reactions that is apparently not determined by endo- versus exonuclease reactions, but best explained by a different orientation of SbcCD during exonuclease and endonuclease reactions with respect to the DNA end. It will be interesting to also analyze eukaryotic homologs for similar activities. What governs such a polarity change in SbcCD is unclear and must await future studies. A possible scenario is that SbcCD can directionally and actively scan DNA, similar to the recently described DNA transport activities of the related SMC proteins (62), until encounter of a blocked end leads to activation of the endonuclease. At an accessible DNA end, the directionality could be reversed through preferential binding to the DNA end, resulting in processive exonuclease. Our comprehensive study sheds light on the cleavage mechanism of the bacterial MR complex and may help to understand the intricate action of its eukaryotic counterpart.

## Supplementary Material

Supplementary Data
