# Supplementary material for: The bacterial Mre11–Rad50 homolog SbcCD cleaves opposing strands of DNA by two chemically distinct nuclease reactions
Source: Nucleic Acids Res. 2018 Oct 2;46(21):11303–14. doi: 10.1093/nar/gky878 (PMC6265447; doi:10.1093/nar/gky878)
Supplement: Supplementary Data [file gky878_supplemental_files.pdf]

Supplementary Data

Supplementary Figure S1

A

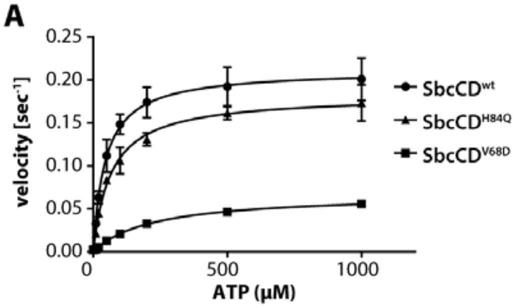

B

	k_{cat} (s^{-1})	K_m (µM)
SbcCD^{wt}	0.212 ± 0.006	46 ± 6
SbcCD^{H84Q}	0.182 ± 0.006	66 ± 8
SbcCD^{V68D}	0.068 ± 0.003	222 ± 25

C

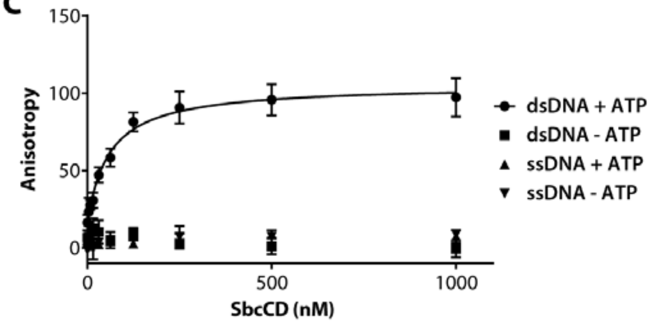

Steady-state ATPase kinetic for SbcCD variants and DNA binding properties of SbcCD^{H84S}

(A) The steady-state ATPase rates of SbcCD variants were tested in the presence of 1 µM 60 bp DNA at and increasing ATP concentrations. The data were fit to a Michaelis-Menten equation, error bars represent the standard deviation of three measurements.

(B) Values were determined by fitting the data of (A) to a Michaelis-Menten equation. Errors represent the standard error of the fit.

(C) DNA binding of SbcCD^{H84S} to 60 nucleotide DNA (ssDNA) and 30 bp DNA (dsDNA) was assayed in the presence of 5 mM $MgCl_2$ and 1 mM $MnCl_2$. DNA concentration was kept at 5 nM and the SbcCD^{H84S} concentration ranged from 2 to 1000 nM. Data points represent the change in fluorescence anisotropy and the data were fit to a 1 to 1 binding equation. Error bars represent the standard deviation from three experiments.

Supplementary Figure S2

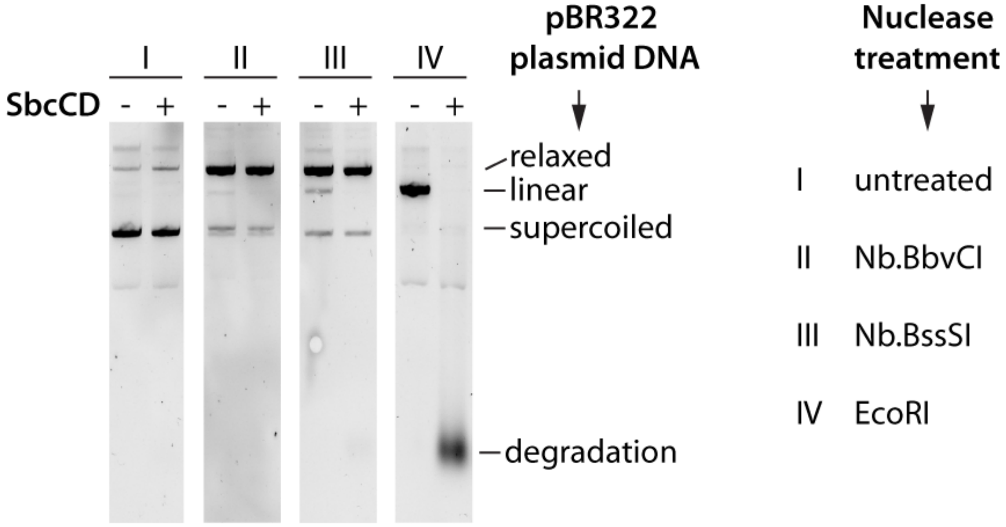

SbcCD^{wt} nuclease activities towards plasmid DNA

SbcCD^{wt} (26 nM) nuclease activity was tested towards pBR322 plasmid DNA (1.7 nM) in the presence of 1 mM ATP, 5 mM $MgCl_2$ and 1 mM $MnCl_2$ at 37 °C for 15 minutes. The pBR322 plasmid was site-specifically incised by the indicated nicking enzymes and EcoRI to create relaxed and linear substrates. Reaction products were separated by agarose gel electrophoresis and stained with GelRed.

Supplementary Figure S3

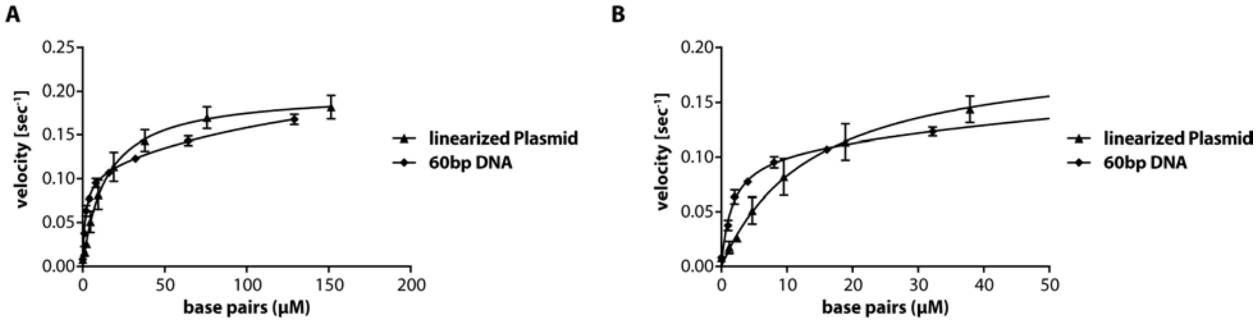

ATP hydrolysis stimulation of the SbcCD^{wt} complex by linear plasmid DNA and 60 bp DNA

(A and B) The ATP hydrolysis rates of SbcCD^{wt} were measured in the presence of 1 mM ATP, 5 mM $MgCl_2$ and 1 mM $MnCl_2$ at 37 °C. Linearized bacteriophage ΦX174 Plasmid DNA (5386 bp in length) and 60 bp DNA were added with different concentrations. ATPase activation by linear plasmid DNA was fit to a Michaelis-Menten equation, activation by 60 bp DNA was fit to a one site and nonspecific binding curve using Prism (GraphPad). Error bars indicate the deviation from three replicates.

Supplementary Table 1

ATP hydrolysis and DNA binding by SbcCD

(A) K_d values were obtained from the anisotropy data in Figure 1C that were fit to a 1 to 1 binding equation. Errors represent the Standard Error of the fit.

(B) Values were determined by fitting the data of the ATP hydrolysis data from Figure 1B to a Michaelis-Menten equation. K_{act} indicates the DNA concentration at half-maximal velocity. Errors represent the Standard Error of the fit.

(C) Kinetic data that were obtained by fitting the data-points of Figure 1A to a Michealis-Menten equation. k_{act} is the plasmid DNA concentration at half-maximum velocity of $SbcCD^{wt}$. Errors represent the Standard Error of the fit.

A

length	K_d (nM)
30 nt	nd
20 bp	800 ± 500
25 bp	150 ± 50
30 bp	43 ± 7
35 bp	57 ± 10
40 bp	52 ± 12
45 bp	59 ± 7
50 bp	57 ± 6

B

length (bp)	K_{act} (nM)	k_{cat} (s^{-1})
20	nd	nd
25	nd	nd
30	1700 ± 700	0.14 ± 0.03
35	280 ± 50	0.120 ± 0.008
40	152 ±19	0.108 ± 0.004
50	66 ± 5	0.117 ± 0.002
60	53 ± 4	0.105 ± 0.002

C

Plasmid	single stranded	supercoiled	nicked	linear
k_{cat} (s^{-1})	0.010 ± 0.001	0.072 ± 0.006	0.21 ± 0.02	0.200 ± 0.007
DNA-Activation	1.3	9	26	25
k_{act} (nM)	nd	4.7 ±1.1	10.8 ± 2.1	2.6 ± 0.3

Supplementary Figure S4

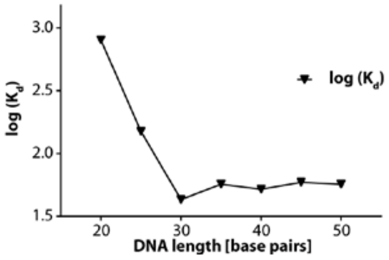

Logarithmic representation of SbcCD's K_D values (from Supplementary Table 1)

Supplementary Figure S5

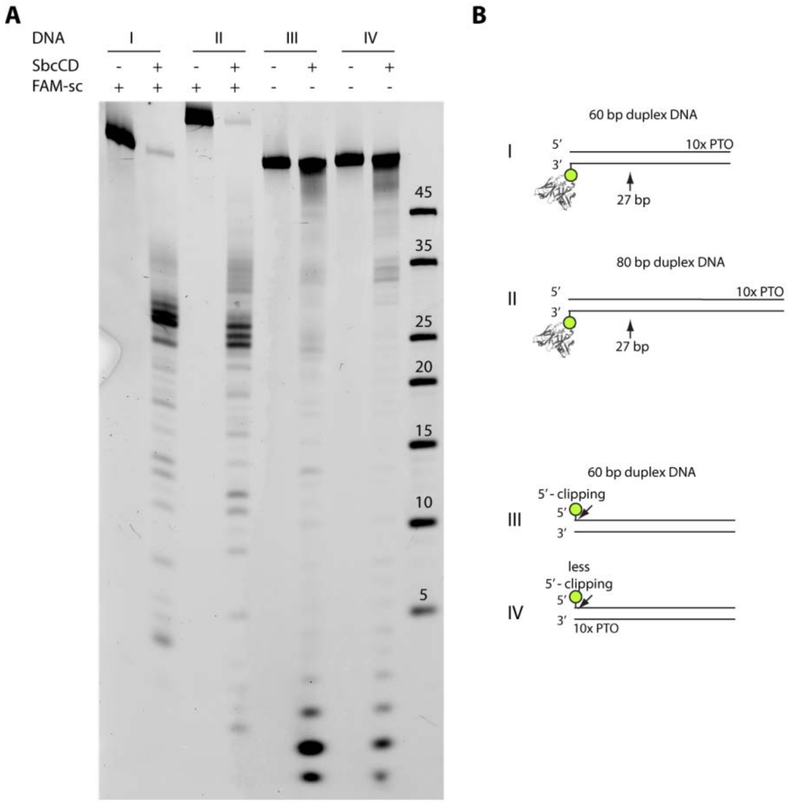

Nuclease activity of SbcCD^{wt} towards 60 and 80 bp DNA and phosphorothioate protected 5′ labeled DNA.

(A) SbcCD^{wt} was assayed in the presence of 1 mM ATP(γS), 5 mM $MgCl_2$ and 1 mM $MnCl_2$ at 37 °C. 60 and 80 bp DNA were protected by a single chain fragment that binds to Fluorescein dyes (FAM-scFv) to generate a protein protein blocked DNA end and assay the endonuclease activity (DNA I and II). Protection of the complementary strand by phosphorothioates (PTO) reduces the 5′ clipping activity by approximately 3-fold.

(B) DNA substrates are schematized that were used in (A).

Supplementary Figure S6

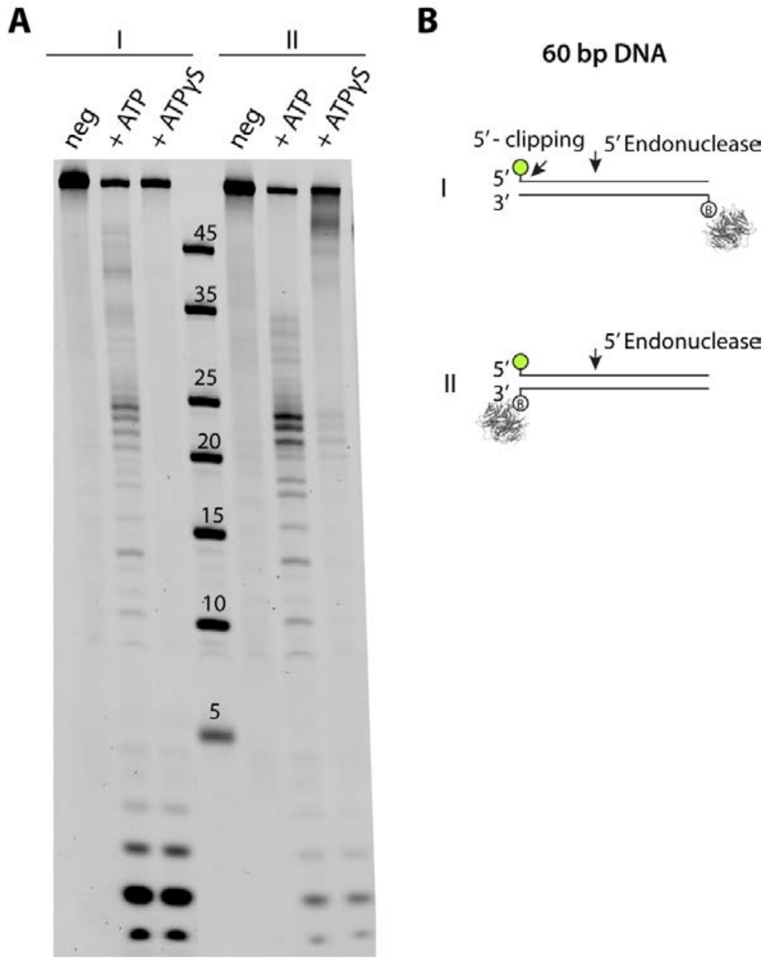

Nuclease activity of SbcCD^{wt} towards 5′ labeled 60 bp DNA

(A) SbcCD^{wt} was assayed in the presence of 1 mM ATP(γS), 5 mM $MgCl_2$ and 1 mM $MnCl_2$ at 37 °C with 5′ labeled 60 bp DNA. The 5′ endonuclease activity remains in the presence of biotin-Streptavidin blocks on the opposite strand

Supplementary Figure S7

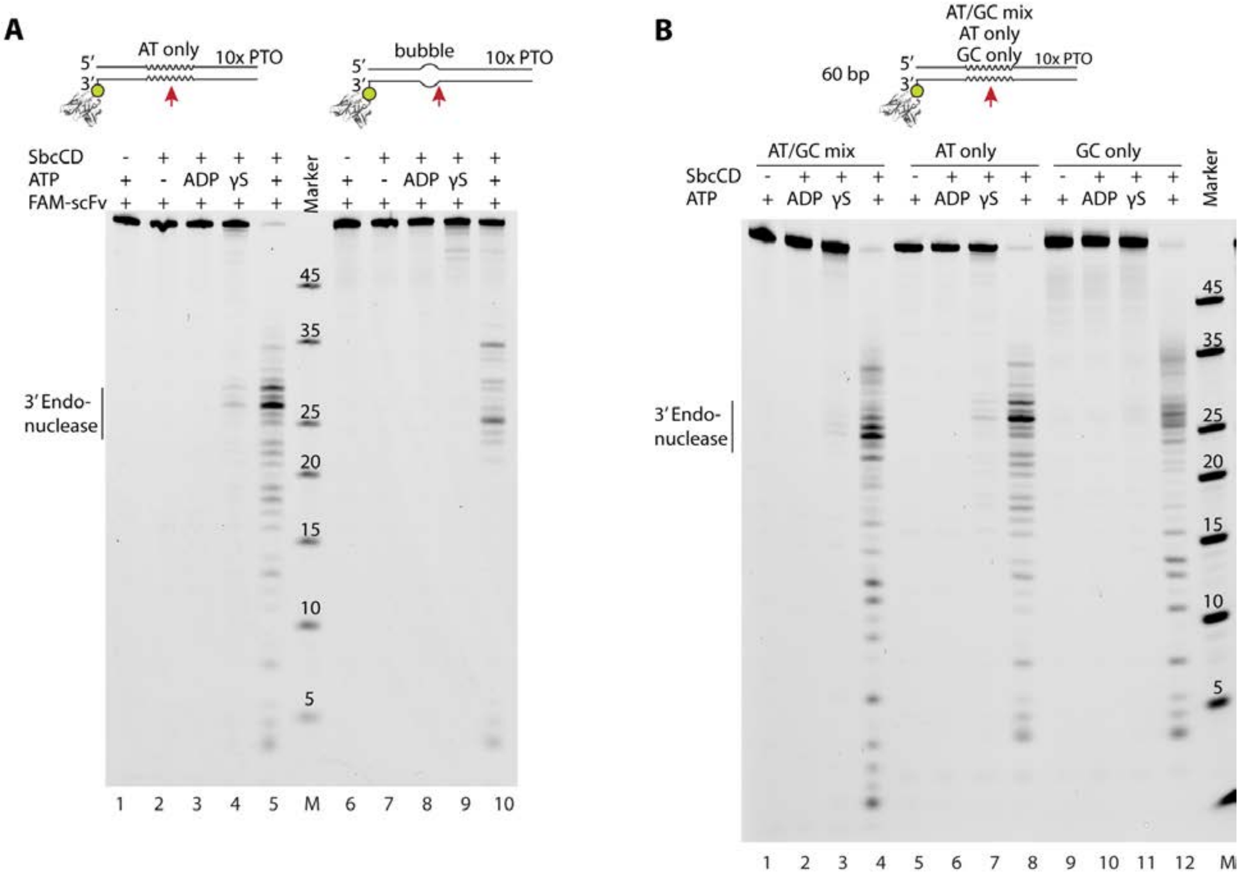

SbcCD^{wt} has endonuclease activity on a protein-blocked 60 bp DNA in the presence of non-hydrolysable ATPγS

(A) SbcCD^{wt} was assayed in the presence of 1 mM ATP(γS) or ADP, 5 mM $MgCl_2$ and 1 mM $MnCl_2$ at 37 °C. SbcCD^{wt} has endonuclease activity on a protein-blocked 60 bp duplex DNA in the presence of ATP and maintains a fractional endonuclease activity with non-hydrolysable ATPγS. On a 60 bp DNA containing a bubble from position 19-23 (relative to the dye) endonuclease activity is observed with ATP but not with ATPγS.

(B) SbcCD^{wt} was assayed as in (A), but with 60 bp DNA that contained different local AT/GC contents from position 15 - 29 relative to the dye. SbcCD^{wt} endonuclease activity has a slight preference for AT-only substrates in the presence of ATPγS.

Supplementary Figure S8

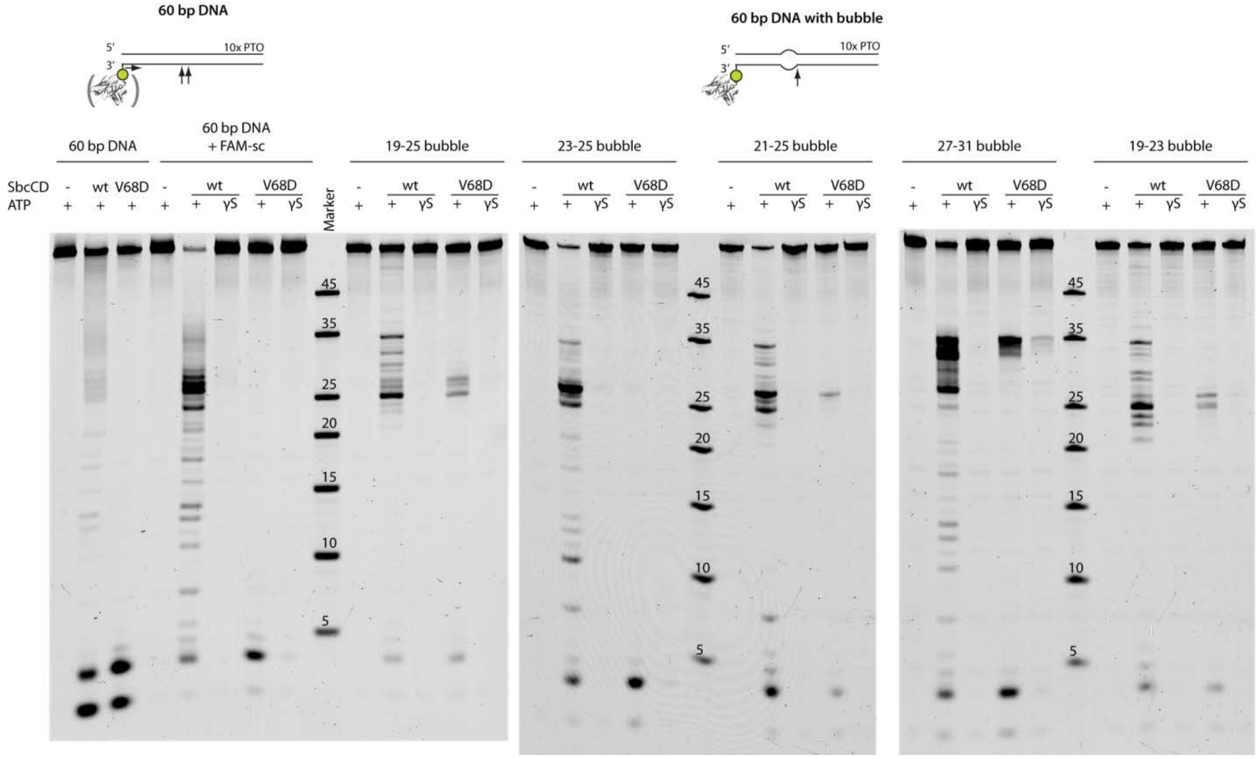

Nuclease activity of SbcCD^{wt} and SbcCD^{V68D} towards 60 bp DNA with pre-melted stretches

SbcCD^{wt} and SbcCD^{V68D} were assayed in the presence of 1 mM ATP(γS), 5 mM $MgCl_2$ and 1 mM $MnCl_2$ at 37 °C. All DNA substrates were 3' labeled with 6-FAM, the complementary strand was protected by 10x phosphorothioate (PTO) linkages to prevent exonucleolytic degradation by SbcCD. The position of the pre-melted stretches are indicated and relative to the dye position.

Supplementary Figure S9

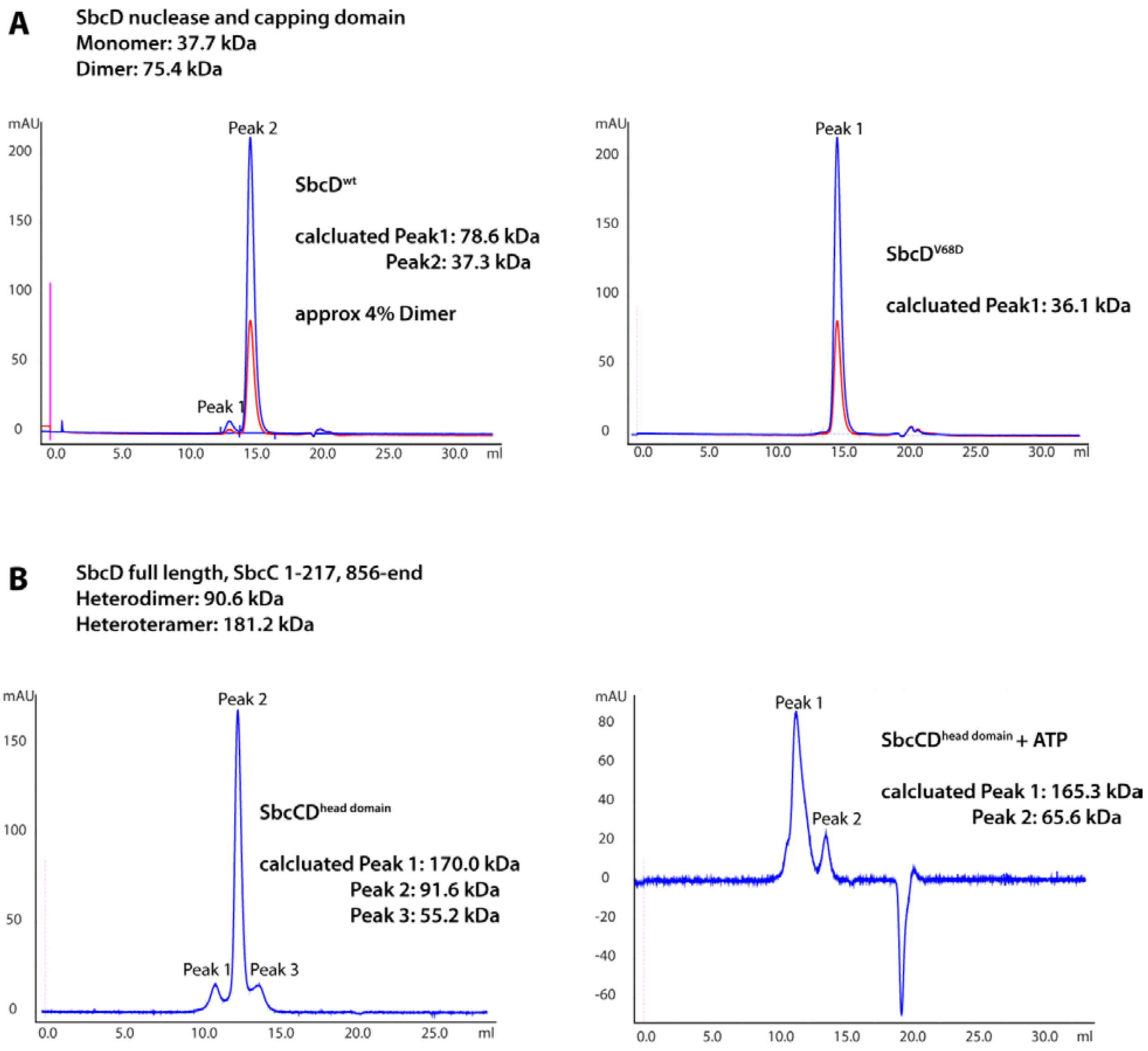

Right angle light scattering (RALS) analysis of SbcD nuclease and capping domain SbcCD head complex

(A) SbcD nuclease and capping domain mainly forms a monomer, 4% of SbcD is a dimer. The SbcD^{V68D}-mutant induced a homogenous monomer of SbcD, therefore, a destabilization of the SbcD interface.

(B) Analysis of SbcD full length in complex with SbcC with shortened coiled-coils (SbcC^{scc}). SbcC^{scc}D mainly forms a heterodimer, 6% is forming a heterotetramer in the absence of ATP (left). The addition of 0.2 mM ATP in the running buffer induces the formation of a SbcCD-head domain heterotetramer (right).

Supplementary Figure S10

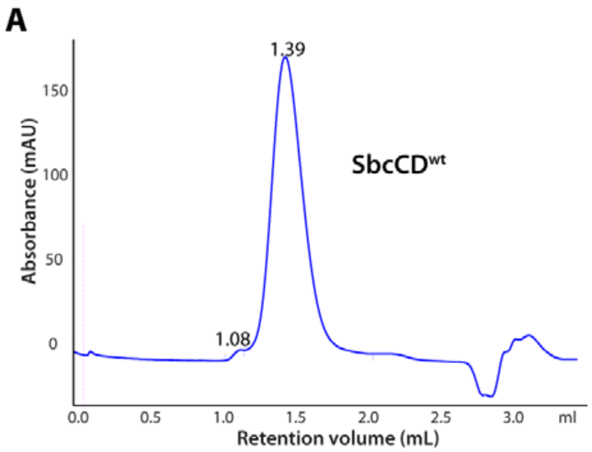

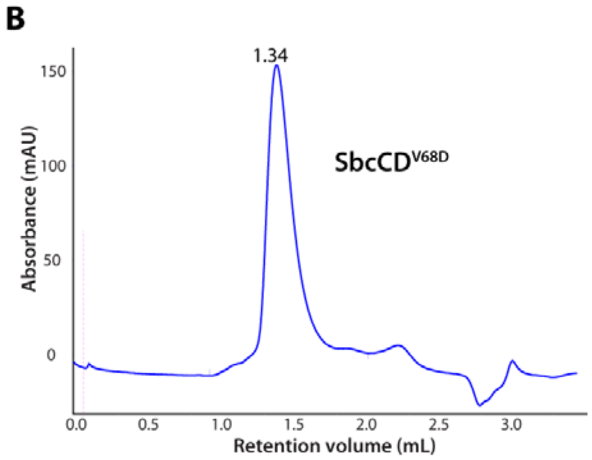

Size-exclusion chromatography analysis of full-length SbcCD variants

(A and B) SbcCD^{wt} and SbcCD^{V68D} were analyzed on a Superose 6 5/150 size-exclusion exclusion column.

Supplementary Figure S11

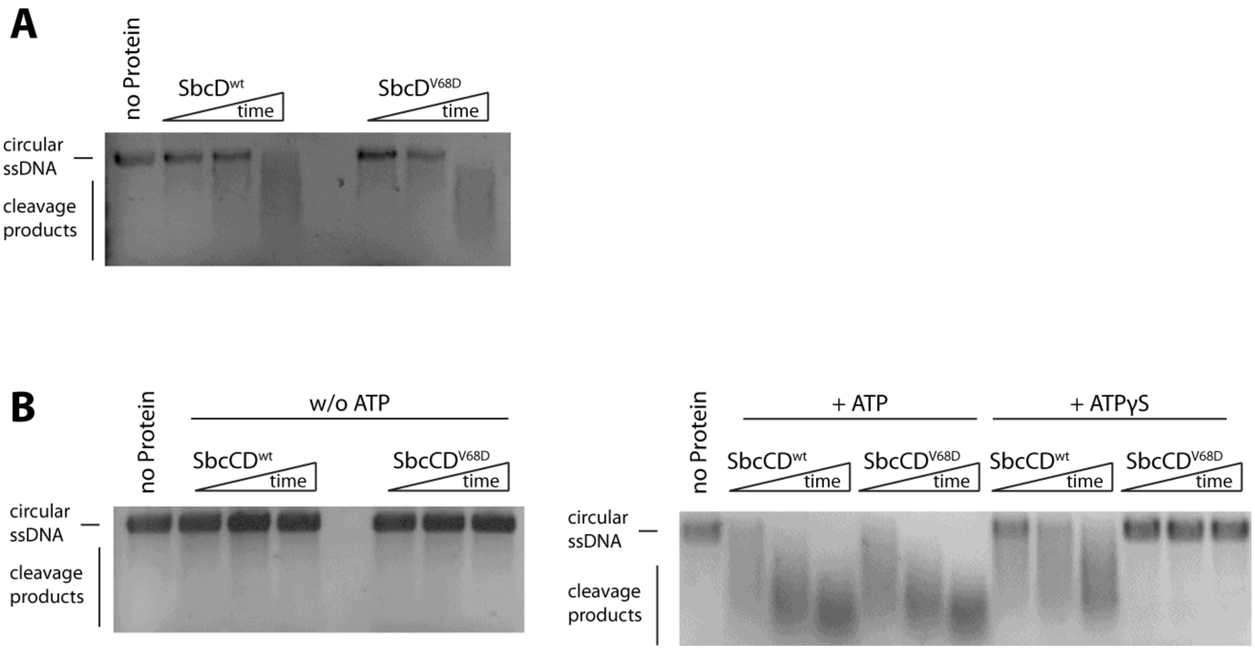

ssDNA endonuclease activity of SbcD and SbcCD dimerization variants

(A) SbcD nuclease and capping domain variants were assayed with ΦX174 Virion DNA in the presence of 1 mM $MnCl_2$ at 37 °C. Samples were taken at 3, 7 and 20 minutes and then quenched. Reaction products were separated by agarose gel electrophoresis and post-stained with GelRed. Destabilizing the SbcD dimer interface does not affect endonuclease activity (top). Formation of a SbcD dimer by LisH enhances endonuclease activity (bottom).

(B) SbcCD dimerization variants were assayed as in (A), dependent to the absence and presence of ATP(γS). $SbcCD^{wt}$ requires ATP binding for ssDNA processing. The presence of ATPγS induces ssDNA processing by $SbcCD^{wt}$ but not by $SbcCD^{V68D}$.

Supplementary Figure S12

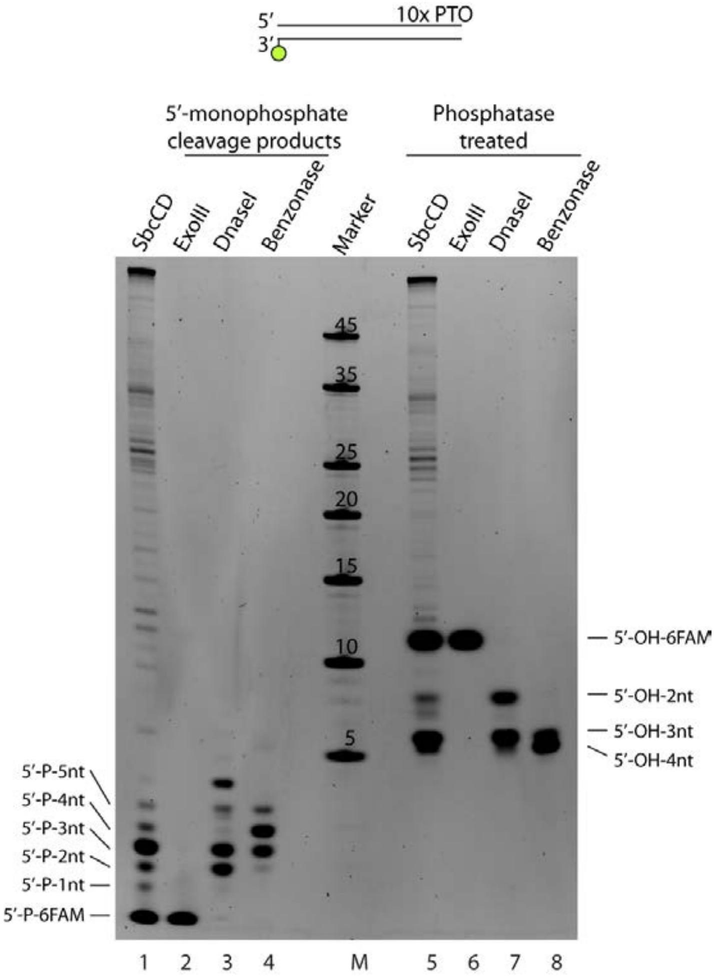

Comparison of DNA cleavage reactions by SbcCD, ExoIII, DNase I and Benzonase®.

SbcCD^{wt} was assayed with 3' 6FAM labeled 60bp DNA under standard nuclease-assay conditions (lane1). To verify 5'-monophosphate of SbcCD's 3'-exonuclease cleavage products, the identical DNA substrate was treated with ExoIII, Dnase I and Benzonase® (lanes 2-4). ExoIII catalyzes the stepwise removal of mononucleotides from 3´ the terminus of duplex DNA and cleaves off the 6FAM-dye (58), Dnase I is an endonuclease that nonspecifically cleaves DNA to release di-, tri- and oligonucleotides (59,60), Benzonase® nonspecifically digests DNA into fragments ranging from 3 to 5 nucleotides (61). These nucleases produce 5'-monophosphorylated and 3'-hydroxylated ends. The nuclease reactions were treated with alkaline phosphatase (lanes 5-8), which induce a migration shift of all short nuclease products and verifies the 5'-monophosphate of SbcCD's 3'-exonuclease cleavage products. These data also show that SbcCD releases the terminal dye, followed by a release of fragements ranging from 1-5nts, with a major trinucleotide species.

Supplementary Figure S13

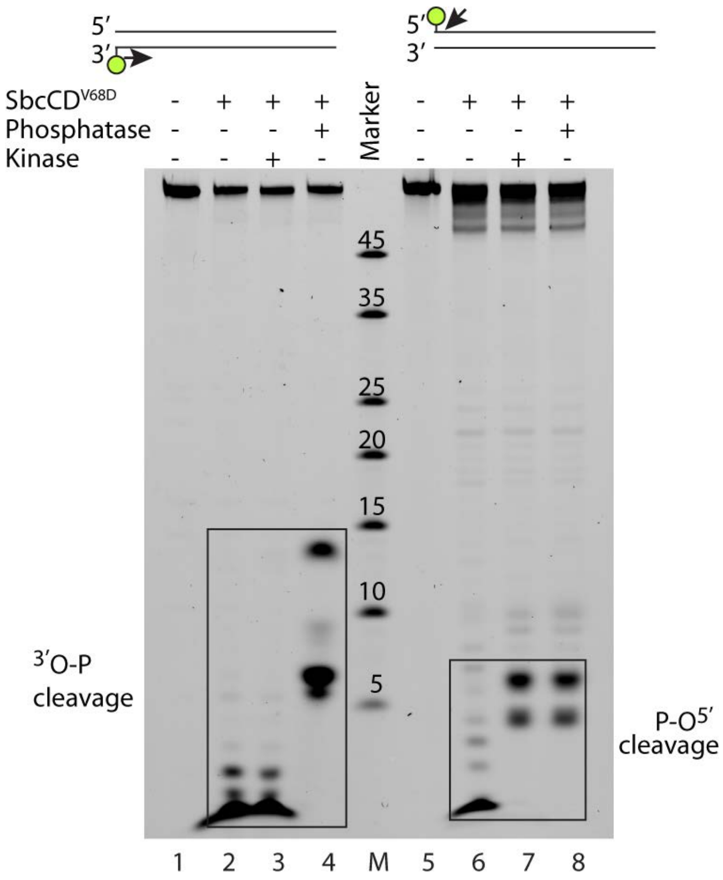

SbcCD^{V68D} has same the exonuclease cleavage-polarity as SbcCD^{wt}

(A) SbcCD^{V68D} was assayed in the presence of 1 mM ATP, 1 mM $MnCl_2$ and 5 mM $MgCl_2$ at 37 °C. The cleavage products of the quenched nuclease reactions were treated with T4 Polynucleotide Kinase or Antarctic Phosphatase to remove or add a phosphate to the DNA ends. The T4 Kinase also catalyzes the removal of 3´-phosphoryl groups from oligonucleotides and therefore induces an identical shift to the Antarctic phosphatase. The altered electrophoretic mobility indicates whether a phosphate is bound to the cleavage products. The mobility-shift pattern of SbcCD^{V68D} is identical to SbcCD^{wt}.
